# The Antidiabetic Activity of* Nigella sativa* and Propolis on Streptozotocin-Induced Diabetes and Diabetic Nephropathy in Male Rats

**DOI:** 10.1155/2017/5439645

**Published:** 2017-02-16

**Authors:** Haddad A. El Rabey, Madeha N. Al-Seeni, Amal S. Bakhashwain

**Affiliations:** ^1^Biochemistry Department, Faculty of Science, King Abdulaziz University, Jeddah, Saudi Arabia; ^2^Bioinformatics Department, Genetic Engineering and Biotechnology Institute, University of Sadat City, Sadat City, Monufia, Egypt

## Abstract

This study was conducted to compare the ameliorative effect of* Nigella sativa* and propolis methanol extract on* streptozotocin*-induced diabetic male rats and treating diabetic nephropathy. Forty male Albino rats were divided into four groups; the first group was the negative control fed standard diet. The other 30 rats were injected with* streptozotocin to induce* diabetes by a single intravenous injection and then divided equally into three groups; the second group was the positive diabetic control; the third and the fourth groups were treated orally with 20% w/w* Nigella sativa *seeds methanol extract and propolis methanol extract (20% w/w), respectively. The rats of the second group showed increased glucose levels and lipid peroxide accompanied with reduction in superoxide dismutase, catalase, and glutathione-S-transferase enzyme activities compared with the negative control. Carboxymethyl lysine, interleukin-6, and immunoglobulins were also increased as a result of diabetes. Kidney function parameters were also elevated, while potassium and sodium levels were decreased. Moreover, tissues of kidney and pancreas showed severe histopathological changes. Treating the diabetic rats with* Nigella sativa* and propolis methanol extract in the third and fourth groups, respectively, ameliorated all altered biochemical and pathological examinations approaching the negative control. Propolis was more effective than* Nigella sativa*.

## 1. Introduction

Diabetes mellitus (DM) is a heterogeneous disease, characterized by chronic hyperglycaemia caused by defects in insulin secretion, insulin action, or both, resulting in impaired function in carbohydrate, lipid, and protein metabolism [1]. Moreover, hyperglycemia is considered a major factor responsible for the intense oxidative stress in diabetes through the overproduction of reactive oxygen species [2, 3], which results in an imbalance between excess formation of reactive oxygen species (ROS) and ability of a biological system to readily detoxify the reactive intermediates or to repair the resulting damage. ROS interact with the free amino and sulfhydryl groups of proteins forming Amadori products which further modified to form advanced glycation end products (AGEs) especially carboxymethyl lysine (CML) [4], contributing to development of diabetic complication [5]. The formed AGEs bind to their receptors (AGE-receptors) on the cell membrane resulting in the activation of the nuclear factor kappa B (NF-*κ*B), which plays an important role in inducing genes involved in the control of the immune system as well as in the response to injury and infection such as IL-6 and immunoglobulins [4].

Effective control of hyperglycemia in diabetic patients is critical for reducing the risk of micro- and macrovascular complications [6]. Natural sources play an important role in the management of diabetes mellitus, especially in developing countries, delaying the development of diabetic complications and correcting the metabolic abnormalities [7].* Nigella sativa* (black seed) and propolis are among the natural sources reported to have beneficial effects in the treatment of many diseases [8].* N. sativa* has many beneficiary effects such as an anticancer, anti-inflammatory, cardiovascular, renal, immunomodulatory, and antidiabetic effects as well as many other effects like antiasthmatic, antimicrobial, antiparasitic, and antihypertensive effects. Moreover, the seeds of* N. sativa* are widely used in the treatment of various diseases like bronchitis, diarrhea, rheumatism, and skin disorders [9]. The efficacy of* N. sativa* is related to numerous active components which have been isolated from seeds and its oil including thymoquinone, thymohydroquinone, dithymoquinone, thymol, carvacrol, nigellimine-N-oxide, nigellicine, nigellidine, and alpha-hederin [10], as well as flavonoids [11].

Propolis is a natural resinous mixture produced by honeybees from substances collected from parts of plants, buds, and exudates which is widely used in folk medicine in various parts of the world for several applications as anti-inflammatory [12], antioxidative [13], antiproliferative [14], antidiabetic [15], and antimicrobial [16] agent. More than three hundred organic compounds of different groups, mainly phenolic, such as flavonoids and phenolic acids, have been identified in propolis [17]. Furthermore, caffeic acid of propolis is known to play an important role in reducing the inflammatory response and also aids the immune system by promoting phagocytic activities and stimulates cellular immunity [18].

This study aimed to evaluate the protective effect of* Nigella sativa* and propolis methanol extracts on streptozotocin-induced diabetes and treating diabetic nephropathy in male rats.

## 2. Materials and Methods

*N. sativa* and propolis were purchased from a local herbal medicine shop in Jeddah, Saudi Arabia.

### 2.1. Diet

The animal diet was obtained from a grain mill in Jeddah, Saudi Arabia. A 100 g of the conventional animal basal diet consists of 4 g corn oil (4% fat), 4 g minerals (4% minerals), 12% protein (17.14 g of 70% casein), 0.2 g choline chloride (0.2%), 0.3 g methionine (0.3%), 4 g cellulose (4% fiber), 1 g vitamin mixture (1% vitamin), and 69.36 g of corn starch (69.36%). The diet was stored in a dark dry place.

Total carotenoids were extracted with acetone-hexane mixture and determined with a spectrophotometer at wavelength of 440 nm as described by Dubois et al. [19].

### 2.2. Preparation of Methanol Extract

Methanol extracts were prepared by soaking 200 g of dry* N. sativa *seeds or propolis in 1 liter of 90% methyl alcohol under shaking for 5 days and then kept in a refrigerator. Methanol was evaporated using a rotatory evaporator apparatus. 20 g of the semisolid extract of both* N. sativa* and propolis was suspended in 100 mL distilled water with 2 mL of tween 80 (suspending agent) to prepare a 20% solution [20].

### 2.3. Phytochemical Analysis

The total flavonoid content of each extract was determined by a colorimetric method as described by Zhishen et al. [21]. 0.5 mL of each sample was mixed with 2 mL of distilled water, and then 0.15 mL of NaNO_2_ solution (15%) was added. 0.15 mL of aluminum chloride (AlCl_3_) solution (10%) was added after 5 m and allowed to stand for 6 minutes, and then 2 mL of 4% NaOH solution was added to the mixture. Water was added to bring the final volume to 5 mL immediately. The mixture was thoroughly mixed and allowed to stand for another 15 minutes. The absorbance of the mixture was measured at 510 nm.

### 2.4. Animals and Housing Conditions

Forty male Albino rats (180–200 g) were obtained from the animal experimental unit of King Fahd Center for Medical Research, King Abdulaziz University. The animal experiments were carried out according to protocols approved by the Institutional Animal House of the University of King Abdulaziz at Jeddah, Saudi Arabia. Rats were kept for two weeks before the start of the experiment for acclimatization. The animals were then housed 5/cage and received normal basal diet and tap water ad libitum at a room temperature of about 28 ± 2°C, a room humidity of 60 ± 5%, and a 12 h light and 12 h dark cycle.

### 2.5. Experiment Design

The animals were divided into 4 groups, each consisting of 10 rats. The first group (G1) received only a single tail vein injection of 0.1 mol/L citrate buffer. The other 30 rats were subjected to fasting for 12 h and were then intravenously injected with freshly prepared streptozotocin (65 mg/kg body weight) in a 0.1 mol/L citrate buffer (pH 4.5). After 5 days of injection, rats with blood glucose higher than 200 mg/dL in the fasting state were considered diabetic. The other rats with blood glucose lower than 200 mg/dL were discarded from the study. The experiments were started one week after STZ injection. The 30 diabetic rats were then randomly divided into 3 groups: the second group (G2) received only STZ and fed normal basal diet. The third group (G3) was treated with (20% w/w)* Nigella sativa* seeds methanol extract using stomach tube. The fourth group (G4) was treated with (20% w/w) propolis methanol extract using stomach tube. Treatment was continued for 4 weeks.

### 2.6. Urine Sample

Urine samples were collected before induction of diabetes and one day before the end of the experiment by placing the rats in individual metabolic cages for 24 h. Albumin and creatinine levels were determined in the urine samples.

### 2.7. Blood Sampling and Analysis

At the end of the experiment, rats were fasted 14–16 hours after their last feeding, and then blood samples were collected from the heart of each rat under anesthesia with diethyl ether. Blood samples were centrifuged at 2,000*g* for 10 minutes at 4°C and serum was removed and stored at −80°C until analysis.

### 2.8. Dissection

The abdomen of ether anaesthetic rats was dissected and the kidneys and pancreas were dissected out. One kidney was kept in ice for kidney homogenate preparation and the other kidney and the pancreas were saved in saline buffer (0.9% NaCl) for histopathological investigations.

### 2.9. Kidney Tissue Homogenate

Kidney tissues were cut into small pieces, washed with phosphate-buffered saline, and then ground in a homogenization buffer consisting of 0.05 M Tris-HCl pH 7.9, 25% glycerol, 0.1 Mm EDTA, and 0.32 M (NH_4_)_2_SO_4_ containing a protease inhibitor tablet (Roche, Germany). The lysates were homogenized on ice using a Polytron homogenizer and then sonicated in an ice bath to prevent overheating for 15 seconds followed by 5-minute centrifugation at 12000 rpm and 4°C. The supernatant was aliquoted and stored at −80°C until use. The kidney homogenate was used for estimating the activity of antioxidant enzymes and lipid peroxidation as well as level of IL-6.

### 2.10. Determination of Fasting Blood Sugar (FBS)

Fasting blood sugar was estimated using glucose kit from HUMAN (Germany) according to Barham and Trinder [22].

### 2.11. Determination of Lipid Peroxide

Lipid peroxide was estimated by determination of malondialdehyde (MDA) in the serum and in the kidney tissue homogenate according to the method described by Ohkawa et al. [23] using commercially available kits from Biodiagnostic Chemical Company (Egypt).

### 2.12. Estimation of Antioxidant Enzymes Activity

Superoxide dismutase (SOD), catalase (CAT), and glutathione-S-transferase (GST) activities were estimated in the serum and in the kidney tissue homogenate according to the method described by Nishikimi et al. [24], Aebi [25], and Habig et al. [26], respectively, using commercially available kits from Biodiagnostic Chemical Company (Egypt).

### 2.13. Determination of Interleukin-6 (IL-6)

IL-6 levels in serum and kidney tissue homogenate were determined using Rat IL-6 Immunoassay kit from R&D Systems Inc. (USA) according to the method of Hibi et al. [27].

### 2.14. Determination of Immunoglobulins (Ig)

Immunoglobulins (IgA, IgM, and IgG) were estimated in the serum according to Fahey and Mckelvey [28] and Berne [29] using commercially available kits from GenWay Biotech (USA).

### 2.15. Determination of Carboxymethyl Lysine (CML)

Carboxymethyl lysine (CML) was estimated in the serum using commercially available kit according to the method described by Seigel et al. [30] from MyBioSource (Canada). This kit employs Double Antibody Sandwich Technique.

### 2.16. Determination of Kidney Functions

Urea, creatinine, and uric acid were estimated in the serum according to the method described by Fawcett and Scott [31], Bartels et al. [32], and Fossati et al. [33], respectively, using enzymatic colorimetric kit from HUMAN (Germany), while albumin was estimated in urine by ELISA according to Sayed [34] using a Nephrat II Albumin Kit (USA) and the concentration of creatinine urine samples was determined by the commercial HUMAN kit (Germany) according to the method of Bartels et al. [32].

### 2.17. Determination of Electrolytes

Sodium (Na^+^) and potassium (K^+^) were estimated in the serum according to Trinder [35] and Terri and Sesin [36], respectively, using HUMAN kits (Germany).

### 2.18. Physiological Evaluations

- Food intake and water consumption were calculated daily.
- Food efficiency ratio (FER), food efficiency ratio percentage (FER%), body weight gain (BWG), and body weight gain percentage (BWG%) were calculated according to the method of Davies and Morris [37].

### 2.19. Histopathological Examination

Kidney and pancreas tissues were kept in saline after animal sacrifice, fixed in 10% formalin, processed routinely, and then embedded in paraffin. 5 *μ*m thick sections were prepared and stained with hematoxylin and eosin (H&E) dye for microscopic investigation (Drury et al. [38]). The stained sections were examined and photographed using Olympus light microscope equipped with a digital camera.

### 2.20. Statistical Analysis

The scored values were analyzed using SPSS program to calculate* t*-test and the mean ± SD and then analyzed using one-way analysis of variance (ANOVA, *P* < 0.05) using a protected least significant difference (LSD) test of SAS [39].

## 3. Results

### 3.1. Phytochemical Analysis

The spectrophotometric evaluation of the antioxidants (flavonoids and carotenoids) showed that* N. sativa* seed contains 993.6 mg/100 g dry weight flavonoids and 80.6 mg/100 g dry weight carotenoids, whereas propolis contains 4630 mg/100 g dry weight flavonoids and 1.92 mg/100 g dry weight carotenoids.

### 3.2. Fasting Blood Sugar (FBS)

Effect of treating STZ-induced diabetic rats with* N. sativa* and propolis for 4 weeks is illustrated in Table 1. The mean values of serum fasting blood sugar (FBS) were significantly (*P* < 0.001) increased in the positive control group, when compared with those of the negative control. However, treating these rats with methanolic extract of* N. sativa* and propolis for 4 weeks significantly (*P* < 0.001) reduced the fasting blood sugar in the serum of both G3 and G4 groups, respectively, although being higher than that of the negative control values. Methanolic extract of propolis in G4 was more effective in reducing fasting blood sugar than that of* N. sativa* in G3.

### 3.3. Lipid Peroxide

Table 1 also shows the effect of treating diabetic rats with methanolic extracts of* N. sativa* and propolis for 4 weeks on lipid peroxidation (MDA) in the serum and kidney tissue homogenate. The mean values of MDA in the diabetic positive control (STZ treated) group were significantly (*P* < 0.001) increased compared with those of the negative control group in both serum and kidney tissue homogenate. In G3 and G4, the mean values of MDA in both serum and kidney tissue homogenate were significantly (*P* < 0.001) decreased compared to those of the positive control group as a result of treating diabetic rats with* N. sativa* and propolis methanolic extract, respectively.

Treating diabetic rats with the methanolic extract of propolis in G4 was more effective on lipid peroxidation compared to that of* N. sativa* in G3.

### 3.4. Antioxidant Enzymes

The results of treating diabetic rats with methanolic extracts of* N. sativa* and propolis for 4 weeks on antioxidant enzymes in the serum and kidney tissue are given in Table 1. The mean values of catalase (CAT), superoxide dismutase (SOD), and glutathione-S-transferase (GST) in the positive control group were significantly (*P* < 0.001) decreased compared to those of the negative control. In G3 and G4, the mean values of CAT, SOD, and GST in the serum were significantly (*P* < 0.001) increased compared to those of the positive control as a result of treating diabetic rats with* N. sativa* and propolis methanolic extract, respectively. In G4, the mean values of the three antioxidant enzymes were higher than those of G3.

### 3.5. Interleukin-6 (IL-6)

Table 2 shows the effect of treating diabetic rats with methanolic extracts of* N. sativa* and propolis for 4 weeks on interleukin-6 (IL-6) in the serum and kidney tissue homogenate. The mean values of IL-6 in the diabetic positive control group (G2) were significantly (*P* < 0.001) increased. After treating these diabetic rats with methanolic extracts of* N. sativa* and propolis in G3 and G4, respectively, a significant (*P* < 0.001) decrease in IL-6 values compared with the positive control group was observed. Treating diabetic rats with propolis in G4 was more effective than treating them with* N. sativa* in G3.

### 3.6. Immunoglobulins (Igs)

The effect of methanolic extracts of* N. sativa* and propolis on IgG, IgA, and IgM immunoglobulins in the serum with induced diabetic rats is given in Table 2. The mean values of IgG, IgA, and IgM immunoglobulins were significantly (*P* < 0.001) increased in the diabetic positive control (STZ treated) compared with those of the negative control group. In G3 and G4, the mean values of IgG, IgA, and IgM immunoglobulins were significantly (*P* < 0.001) decreased as a result of treatment with* N. sativa* and propolis methanolic extract, respectively. The immunoglobulins result revealed that treating STZ-induced diabetic rats with the propolis methanol extract in G4 was more efficient than treating them with the methanolic extract of* N. sativa* in G3.

### 3.7. Carboxymethyl Lysine (CML)

The percentage of CML in the diabetic positive control group (G2) was significantly increased compared with negative control as shown in Table 2. In G3 and G4, the percentage of CML was very highly significantly (*P* < 0.001) decreased as a result of treating diabetic rats with* N. sativa* and propolis, respectively, compared with the positive control group. In G4, treating diabetic rats with methanolic extract of propolis was more effective on CML compared to treating them with* N. sativa* in G3.

### 3.8. Kidney Functions

The mean values of urea, creatinine, and uric acid in the serum of the positive control group (G2) were significantly (*P* < 0.001) increased compared with those of the negative control group (G1) as a result of induced diabetes shown in Table 3. Treating these diabetic rats with methanolic extracts of* N. sativa* and propolis in G3 and G4, respectively, significantly (*P* < 0.001) decreased urea, creatinine, and uric acid levels compared with those of the positive control group (G2). The methanolic extract of propolis in G4 was more effective than that of* N. sativa* in G3.

Also, Table 3 shows that the mean values of urinary albumin of the positive control group were significantly (*P* < 0.001) increased compared with those of the negative control group (G1). Meanwhile, the mean values of urinary creatinine in G2 were significantly (*P* < 0.001) decreased compared with those of the negative control group (G1). Treating these diabetic rats in G3 and G4 with methanolic extract of* N. sativa* and propolis, respectively, significantly (*P* < 0.001) decreased urinary albumin and increased creatinine in urine when compared with those of the positive control (G2).

### 3.9. Serum Electrolytes

Table 3 also shows the effect of treating diabetic rats with methanolic extracts of* N. sativa* and propolis for 4 weeks on serum electrolytes. The mean values of serum sodium and potassium ions of the positive group were significantly (*P* < 0.001) decreased compared with those of the negative control group. Treating these diabetic rats with methanolic extracts of* N. sativa* and propolis in G3 and G4, respectively, significantly (*P* < 0.001) increased serum electrolytes levels (Na^+^ and K^+^) compared with those of the positive control group.

### 3.10. Food Intake

Table 4 shows that there were nonsignificant differences in food intake (FI) in all groups in the first and the second week, whereas the mean values of FI in all groups in the 3rd week were significantly (*P* < 0.01) lower than those of the negative control. In the fourth, FI of the positive control group was significantly (*P* < 0.05) lower than that of the negative control group. In G3 and G4, FI was nonsignificantly lower than that of the positive control group.

### 3.11. Water Consumption

Data in Table 5 illustrate the effect of supplementation of methanolic extract of* N. sativa* and propolis for 4 weeks to diabetic rats on water consumption. The positive control group showed significant (*P* < 0.001) increase in water consumption in the first three weeks as a result of STZ-induced diabetes, compared with that of the negative control group, whereas the 4th week showed no significant difference in water consumption in all groups. Treating diabetic rats with* N. sativa* and propolis significantly (*P* < 0.001) decreased water consumption, compared with that of positive group.

### 3.12. Physiological Evaluation

Table 6 shows the effect of treating STZ-induced diabetic rats with* N. sativa* and propolis for 4 weeks on physiological evaluation. The mean values of body weight gain (BWG), body weight gain percentage (BWG%), food efficiency ratio (FER), and food efficiency ratio percentage (FER%) in the positive control group were nonsignificantly lower than those of the negative control. Treating these diabetic rats with methanolic extract of* N. sativa* in G3 significantly (*P* < 0.01) decreased the mean values of these parameters when compared with the positive group, whereas the mean values of these parameters in G4 were nonsignificantly lower than those of the positive control group.

### 3.13. Pathology of Kidney

**Figure 1:**
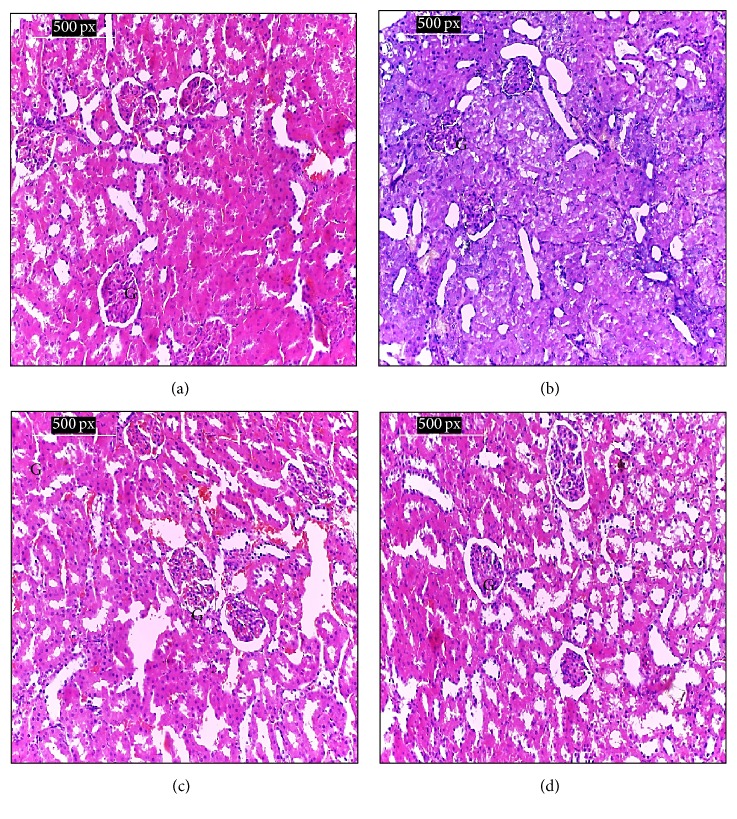
(a) Kidney of rats of the negative control group reveals the normal histological structure of renal tissue with normal parenchyma, normal blood vessels, and being interstitial with no histopathological changes, (b) kidney of rat from the positive control group showing collapsed glomerular tuft with marked tubular atrophy, interstitial inflammation, and interstitial hemorrhage, (c) kidney of diabetic rat treated with* N. sativa* methanol extract showing normal glomeruli and regenerated tubules with interstitial hemorrhage, and (d) kidney of diabetic rat treated with propolis methanol extract (G4) showing near normal renal cortical tissue. G: glomerulus (H&E stain ×200).

Microscopically, the histopathological examination of the kidney of the negative control group showed normal histological structure of normal kidney tissues and normal blood vessels with no histopathological changes (Figure 1(a)). Examination of kidney tissues of rats in the positive group, which suffer from diabetes, showed pathological changes in kidney structure compared to that of the negative control group. They showed a collapsed glomerular tuft with marked tubular atrophy associated with interstitial inflammation and interstitial hemorrhage (Figure 1(b)). Meanwhile, the kidney sections of diabetic rats in G3 treated with the* N. sativa* methanol extract for 4 weeks seemed to be restoring the normal appearance of glomeruli and regenerated tubules with interstitial hemorrhage (Figure 1(c)). On the other hand, after treatment with the propolis methanol extract in group (4) for 4 weeks, the kidney nearly restored the normal cortical tissue (Figure 1(d)).

### 3.14. Pathology of Pancreas

**Figure 2:**
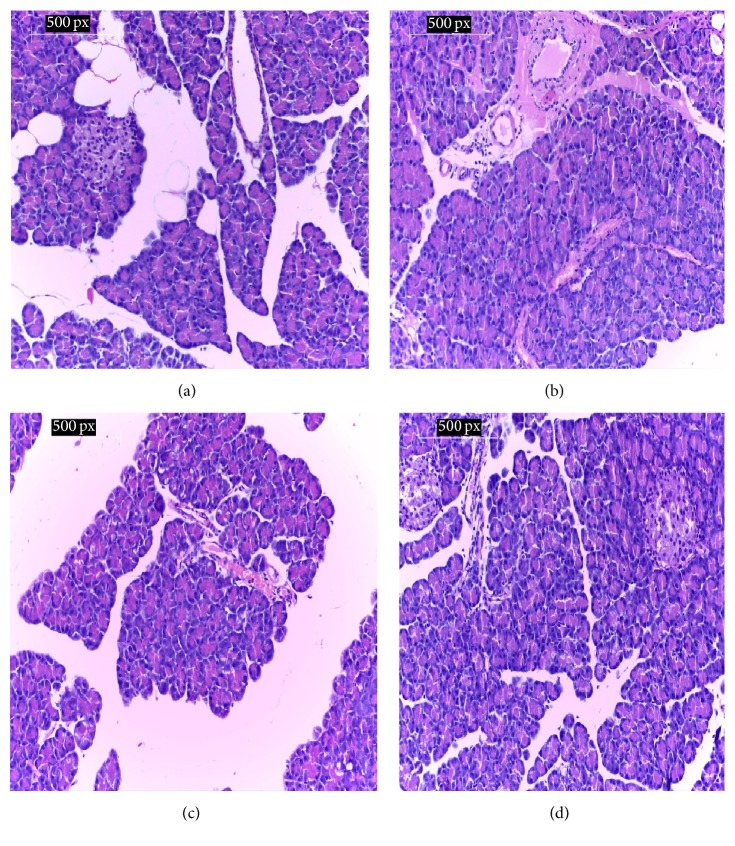
(a) Pancreas of control negative group showing normal pancreatic acini, Langerhans cells, and interductal glands. (b) Pancreas of control positive group showing mild degeneration of pancreatic acini cells with periductal inflammation, edema, and congestion. (c) Pancreas of* Nigella sativa* treated group showing improvement and degeneration of pancreatic tissues with nearly normal tissues. (d) Pancreas of propolis treated group showing restored pancreatic tissues to the normal with no evidence of inflammation in islets or around the large ducts. (H&E, ×200).

Figure 2 shows the histology of pancreas of rats under study.  Figure 2(a) shows the normal pancreatic tissues of the negative control group (G1) with normal pancreatic acini, Langerhans cells, and interductal glands.  Figure 2(b) shows the pancreatic tissues of the streptozotocin-induced diabetic rats of the positive control (G2) with degenerated pancreatic acini cells, periductal inflammation, and mild congested edema.  Figure 2(c) shows the pancreas of diabetic rats treated with* Nigella sativa* (G3) with improvement in the degenerated pancreatic acini cells, mild inflammation, and congestion.  Figure 2(d) shows the pancreas of diabetic rats of the propolis treated group (G4) with no evidence of inflammation in islets or around the large ducts with normal pancreatic tissues.

## 4. Discussion

Diabetes mellitus is metabolic disorder leading to hyperglycemia, which later develops to micro- and macrovascular complications. The induction of experimental diabetes in the rats using chemicals which selectively destroy pancreatic *β*-cells is very convenient and simple to use as streptozotocin (STZ) that acts as diabetogenic agent mediated by reactive oxygen species [40]. In the present study, induction of diabetes using streptozotocin (STZ) at a dose of 65 mg/kg in rats of the positive control group showed significant increase in serum glucose level compared with the control group [3, 41]. The concurrent oral administration of 20% of methanolic extract of* N. sativa* or propolis to the diabetic rats of G3 and G4, respectively, for 4 weeks significantly decreased glucose levels most probably due to their antioxidant chemical contents [42, 43].

STZ diabetic rats in G2 also showed an increase in lipid peroxidation level accompanied by decreased CAT, SOD, and GST activity in the serum and the kidney tissue homogenate compared with that of the negative control group after 4 weeks. This result is in agreement with previous investigations [3, 4, 44]. This result may be attributed to the fact that the elevated generation of free radicals resulting in the consumption of antioxidant defense components may lead to disruption of cellular functions and oxidative damage to membranes and may enhance susceptibility to lipid peroxidation [45]. The concurrent treatment with methanolic extract of* N. sativa* and propolis ameliorated these parameters and nearly restored them to their normal levels as a result of their antioxidant activity due to their contents of phenolics and flavonoids that have scavenging effect on the free radicals [11, 46].

Advanced glycation end products (AGEs) increase reactive oxygen species formation and impair antioxidant systems that activate NF-*κ*B signaling pathway which enhanced the production of the cytokine interleukin-6 (IL-6) involved not only in inflammation and infection responses but also in the regulation of metabolic, regenerative, and neural processes [47]. In the current study, IL-6 was increased in the serum and kidney tissue in positive group as a result of STZ-induced diabetes. A similar result was reported by Sayed [34]. However, IL-6 was decreased with the concurrent treatment with methanolic extract of* N. sativa* and propolis. This result is consistent with Bashandy et al. [48] and Al Ghamdi et al. [49].

The increase of immunoglobulins (IgG, IgA, and IgM) as a result of diabetes in the current study is consistent with the increase in IL-6 and other findings revealed a positive correlation between these parameters [3]. This may be attributed to the fact that production of proinflammatory cytokines is increased in patients with diabetes. These include adipocytokines such as interleukin-6 (IL-6), which is a cofactor for immunoglobulin synthesis and a common marker of inflammation [50]. Treating diabetic rats with* N. sativa* and propolis has significantly reduced immunoglobulins (IgG, IgA, and IgM). These results are consistent with previous studies [51, 52].

Diabetes is associated with severe acute and chronic complications that negatively influence both the quality of life and survival of affected individuals [53]. Therefore, protein glycation and formation of AGEs play an important role in the pathogenesis of diabetic complications like retinopathy, cataract, neuropathy, nephropathy, and cardiomyopathy [54]. N*ε*-(Carboxymethyl) lysine (CML) was selected as a marker of AGEs in laboratory studies. In our study, CML showed significant increase in the untreated diabetic group after 4 weeks compared with the negative control. This result agrees with that of Van Eupen et al. [5]. On the other hand, our result showed that carboxymethyl lysine (CML) was significantly decreased in the treated diabetic group with* N. sativa* and propolis in G3 and G4, respectively, compared with the untreated diabetic group G2. It is worthy to mention that the efficacy of propolis on lowering carboxymethyl lysine (CML) exceeded that of* N. sativa*.

STZ administration increased serum renal markers in rats, for example, creatinine, urea, and uric acid [3, 55], as a result of diabetic nephropathy which is considered a major complication of diabetes [34]. The current investigation is consistent with the previous studies. Furthermore, overtime diabetic nephropathy will be developed which is characterized by proteinuria, a loss of renal function, and a rapid progression to end stage renal failure [7]. Urine analysis of the STZ-induced diabetes rats showed significant increase in albumin level in the positive control group and decreased urinary creatinine level in G2 after 4 weeks. These results are consistent with other studies [3, 56]. Treatment with* N. sativa* and propolis as natural resources showed significant decrease in the levels of urea, creatinine, and uric acid compared with the positive control. The present results were in conformity with the results of Abdelaziz and Kandeel [57] and Saleh [58]. On other hand, oral administration of propolis revealed a significant reduction of urinary albumin and restoring urinary creatinine approaching the negative control level more than treatment with* N. sativa*. The effect of treatment with 20% of methanolic extract of propolis may be attributed to a strong antioxidant effect of propolis, which can ameliorate oxidative stress and delay the occurrence of diabetic nephropathy in diabetes mellitus [59, 60]. The combination of intracellular and extracellular electrolyte disturbances may be implicated in the pathogenesis of neuropathy, nephropathy, and vascular complications in diabetic patients [61]. Our experiment recorded a significant decrease in serum sodium and potassium in the untreated diabetic group. This result agrees with previous investigation of Al-Rubeaan et al. [62] and Liamis et al. [63]. Diabetic rats treated with propolis restored serum electrolyte (Na^+^ and K^+^) levels to normal compared to* N. sativa*.

The renal and pancreatic tissues were severely affected in hyperglycemic rats of G2 as a result of STZ supplementation. This result which is consistent with other studies showed a correlation between hyperglycemia and pathological alteration of vital organs [64, 65]. The concurrent supplementation with* N. sativa* and propolis in G3 and G4, respectively, has significantly improved renal and pancreatic tissues and nearly restored them to their normal state [66, 67].

However, administration of 20% (w/w) methanolic extract of either* N. sativa* or propolis to STZ-induced diabetic rats significantly reduced hyperglycemic and oxidative stress resulting from hyperglycemia. Also, they improved all adverse biochemical and histopathological changes resulting from diabetes. These natural resources revealed safe and excellent antidiabetic activity attributed to their antioxidant activity. As well as overcoming most of the histopathology changes in kidney and pancreas tissues, the majority of the cells restored the normal conditions. In addition, methanolic extract of propolis appeared to be more efficient than* N. sativa* as revealed by the different biochemical and histological investigations. Therefore, it is recommended that dietary* N. sativa* and propolis could be excellent adjuvant support in the therapy of diabetes mellitus and preventing its complications.

## Figures and Tables

**Table 1:** Effect of treating diabetic rats with methanolic extracts of *N. sativa* and propolis for 4 weeks on fasting blood sugar, lipid peroxide, and antioxidants enzymes.

Parameters	Statistics	G1 N. control	G2 P. control	G3 *Nigella sativa *extract	G4 Propolis extract
Serum FBS (mL/dL)	Mean ± SE	92.66 ± 1.14^d^	283.33 ± 2.47^a^	203.16 ± 3.71^b^	139.00 ± 1.18^c^
	LSD				
	0.05 = 7.230				
	*t*-test	—	−63.63^*∗∗∗*^	15.32^*∗∗∗*^	59.03^*∗∗∗*^

Serum MDA (nmol/mL)	Mean ± SE	0.93 ± 0.03^d^	4.50 ± 0.05^a^	2.82 ± 0.03^b^	1.94 ± 0.03^c^
	LSD				
	0.05 = 0.149				
	*t*-test	—	−52.66^*∗∗∗*^	20.35^*∗∗∗*^	27.91^*∗∗∗*^

MDA (nmol/g) Kidney tissue	Mean ± SE	2.58 ± 0.06^d^	16.08 ± 0.18^a^	4.76 ± 0.05^b^	3.54 ± 0.11^c^
	LSD				
	0.05 = 0.306				
	*t*-test	—	−70.12^*∗∗∗*^	67.64^*∗∗∗*^	62.91^*∗∗∗*^

Serum CAT (U/mL)	Mean ± SE	2.40 ± 0.19^a^	0.15 ± 0.01^d^	1.20 ± 0.01^c^	1.89 ± 0.02^b^
	LSD				
	0.05 = 0.301				
	*t*-test	—	11.49^*∗∗∗*^	−71.53^*∗∗∗*^	−58.72^*∗∗∗*^

Serum SOD (U/mL)	Mean ± SE	638.68 ± 1.56^a^	120.83 ± 2.41^d^	276.45 ± 2.37^c^	520.18 ± 1.85^b^
	LSD				
	0.05 = 25.419				
	*t*-test	—	178.65^*∗∗∗*^	−48.94^*∗∗∗*^	−116.79^*∗∗∗*^

Serum GST (U/mL)	Mean ± SE	813.20 ± 2.32^a^	120.93 ± 2.38^d^	421.56 ± 3.20^c^	762.65 ± 1.74^b^
	LSD				
	0.05 = 6.450				
	*t*-test	—	228.70^*∗∗∗*^	−136.08^*∗∗∗*^	−275.19^*∗∗∗*^

CAT (U/g) Kidney tissue	Mean ± SE	5.02 ± 0.08^a^	0.385 ± 0.02^c^	2.86 ± 0.03^d^	3.97 ± 0.06^b^
	LSD				
	0.05 = 0.144				
	*t*-test	—	51.41^*∗∗∗*^	−74.97^*∗∗∗*^	−56.95^*∗∗∗*^

SOD (U/g) Kidney tissue	Mean ± SE	917.18 ± 2.59^a^	175.58 ± 4.53^d^	675.98 ± 3.94^c^	818.73 ± 4.78^b^
	LSD				
	0.05 = 12.818				
	*t*-test	—	117.11^*∗∗∗*^	−134.00^*∗∗∗*^	−88.10^*∗∗∗*^

GST (U/g) Kidney tissue	Mean ± SE	826.20 ± 2.75^a^	315.68 ± 3.56^c^	684.33 ± 1.99^b^	771.88 ± 2.69^ab^
	LSD				
	0.05 = 181.965				
	*t*-test	—	109.14^*∗∗∗*^	−71.07^*∗∗∗*^	−103.53^*∗∗∗*^

Data are represented as mean ± SE. *t*-test values: *∗∗∗*: significant at *P* < 0.001. ANOVA analysis: within each row, means with different superscript (a, b, c, or d) are significantly different at *P* < 0.05, whereas means with the same superscript letters mean that there is no significant difference at *P* < 0.05. LSD: least significant difference; NS: nonsignificant.

**Table 2:** Effect of treating diabetic rats with methanolic extracts of *N. sativa* and propolis for 4 weeks on immunoglobulins and IL-6.

Parameters	Statistics	G1 N. control	G2 P. control	G3 *Nigella sativa *extract	G4 Propolis extract
Serum IgG (mg/dL)	Mean ± SE	530.66 ± 1.05^b^	754.33 ± 3.46^a^	595.00 ± 100.64^b^	572.33 ± 2.40^b^
	LSD				
	0.05 = 152.870				
	*t*-test	—	−63.91^*∗∗∗*^	1.55^NS^	45.12^*∗∗∗*^

Serum IgA (mg/dL)	Mean ± SE	99.16 ± 1.88^d^	359.83 ± 1.74^a^	257.00 ± 1.73^b^	126.00 ± 1.31^c^
	LSD				
	0.05 = 5.492				
	*t*-test	—	−85.42^*∗∗∗*^	52.10^*∗∗∗*^	92.71^*∗∗∗*^

Serum IgM (mg/dL)	Mean ± SE	129.83 ± 1.07^d^	357.16 ± 2.24^a^	220.00 ± 2.22^b^	141.50 ± 1.78^c^
	LSD				
	0.05 = 5.614				
	*t*-test	—	−138.06^*∗∗∗*^	45.31^*∗∗∗*^	65.29^*∗∗∗*^

Serum IL-6 (pg/mL)	Mean ± SE	5.60 ± 0.26^d^	24.48 ± 0.89^a^	11.90 ± 0.34^b^	8.78 ± 0.19^c^
	LSD				
	0.05 = 1.630				
	*t*-test	—	−17.24^*∗∗∗*^	11.26^*∗∗∗*^	20.71^*∗∗∗*^

IL6 (pg/g) Kidney tissue	Mean ± SE	48.80 ± 2.01^d^	90.43 ± 1.55^a^	67.01 ± 0.69^b^	55.43 ± 0.89^c^
	LSD				
	0.05 = 4.285				
	*t*-test	—	−14.14^*∗∗∗*^	16.67^*∗∗∗*^	19.38^*∗∗∗*^

Carboxymethyl lysine (CML) (nmol/mL)	Mean ± SE	188.16 ± 2.38^d^	276.00 ± 2.58^a^	234.33 ± 1.85^b^	212.16 ± 2.35^c^
	LSD				
	0.05 = 6.574				
	*t*-test	—	−24.84^*∗∗∗*^	10.93^*∗∗∗*^	23.52^*∗∗∗*^

Data are represented as mean ± SE. *t*-test values superscripts (a, b, c, or d) are significantly different at *P* < 0.05, whereas means with the same superscript letters mean that there is no significant difference at *P* < 0.05. LSD: least significant difference; NS: nonsignificant.

**Table 3:** Effect of treating diabetic rats with methanolic extracts of *N. sativa *and propolis for 4 weeks on kidney functions and electrolytes.

Parameters	Statistics	G1 N. control	G2 P. control	G3 *N. sativa *extract	G4 Propolis extract
Serum urea (mg/dL)	Mean ± SE	24.50 ± 1.11^d^	74.83 ± 0.87^a^	47.33 ± 0.88^b^	33.33 ± 0.98^c^
	LSD				
	0.05 = 2.705				
	*t*-test	—	−29.16^*∗∗∗*^	25.28^*∗∗∗*^	27.26^*∗∗∗*^

Serum creatinine (mg/dL)	Mean ± SE	0.68 ± 0.03^d^	3.63 ± 0.18^a^	2.60 ± 0.09^b^	1.21 ± 0.04^c^
	LSD				
	0.05 = 0.318				
	*t*-test	—	−14.90^*∗∗∗*^	6.70^*∗∗∗*^	11.66^*∗∗∗*^

Serum uric acid (mg/dL)	Mean ± SE	3.33 ± 0.08^d^	6.68 ± 0.04^a^	5.15 ± 0.07^b^	4.20 ± 0.05^c^
	LSD				
	0.05 = 0.179				
	*t*-test	—	−59.53^*∗∗∗*^	31.01^*∗∗∗*^	28.46^*∗∗∗*^

Urinary albumin (mg/dL)	Mean ± SE	22.16 ± 1.70^d^	411.50 ± 7.74^a^	216.66 ± 3.71^d^	122.66 ± 3.27^b^
	LSD				
	0.05 = 14.333				
	*t*-test	—	−47.24^*∗∗∗*^	20.85^*∗∗∗*^	29.03^*∗∗∗*^

Urinary creatinine (mg/dL)	Mean ± SE	85.00 ± 0.85^a^	27.00 ± 0.36^c^	35.16 ± 1.16^b^	73.16 ± 0.60^c^
	LSD				
	0.05 = 2.405				
	*t*-test	—	84.90^*∗∗∗*^	−7.57^*∗∗∗*^	−70.58^*∗∗∗*^

Serum Na^+^ (mmol/L)	Mean ± SE	143.83 ± 0.94^a^	118.33 ± 0.88^d^	127.83 ± 0.60^c^	138.66 ± 0.42^b^
	LSD				
	0.05 = 1.996				
	*t*-test	—	19.85^*∗∗∗*^	−8.99^*∗∗∗*^	−24.11^*∗∗∗*^

Serum K^+^ (mmol/L)	Mean ± SE	4.86 ± 0.03^a^	3.03 ± 0.08^d^	3.76 ± 0.04^c^	4.25 ± 0.04^b^
	LSD				
	0.05 = 0.169				
	*t*-test	—	17.39^*∗∗∗*^	−10.25^*∗∗∗*^	−13.37^*∗∗∗*^

Data are represented as mean ± SE. *t*-test values: *∗∗∗*: significant at *P* < 0.001. ANOVA analysis: within each row, means with different superscript (a, b, c, or d) are significantly different at *P* < 0.05, whereas means with the same superscript letters mean that there is no significant difference at *P* < 0.05. LSD: least significant difference; NS: nonsignificant.

**Table 4:** Effect of treating diabetic rats with methanolic extracts of *N. sativa* and propolis for 4 weeks on food intake.

Food intake (g/day)	Statistics	G1 N. control	G2 P. control	G3 *N. sativa *extract	G4 Propolis extract
1st week	Mean ± SE	15.50 ± 0.22^a^	15.50 ± 0.22^a^	15.50 ± 0.22^a^	15.50 ± 0.22^a^
	LSD				
	0.05 = 0.674				
	*t*-test	—	0.00^NS^	0.00^NS^	0.00^NS^

2nd week	Mean ± SE	16.50 ± 0.22^a^	16.33 ± 0.21^a^	16.66 ± 0.21^a^	16.50 ± 0.22^a^
	LSD				
	0.05 = 0.648				
	*t*-test	—	0.54^NS^	−1.58^NS^	−0.54^NS^

3rd week	Mean ± SE	19.16 ± 0.54^a^	18.16 ± 0.40^b^	17.00 ± 0.44^c^	15.50 ± 0.22^c^
	LSD				
	0.05 = 0.933				
	*t*-test	—	3.87^*∗∗*^	2.90^*∗∗*^	6.32^*∗∗∗*^

4th week	Mean ± SE	16.41 ± 0.39^a^	16.12 ± 0.30^ab^	15.95 ± 0.23^b^	15.91 ± 0.24^b^
	LSD	—	1.77^*∗*^	0.81^NS^	1.15^NS^
	0.05 = 0.426				
	*t*-test				

Data are represented as mean ± SE. *t*-test values: *∗*: significant at *P* < 0.05, *∗∗*: significant at *P* < 0.01, and *∗∗∗*: significant at *P* < 0.001. ANOVA analysis: within each row, means with different superscript (a, b, c, or d) are significantly different at *P* < 0.05, whereas means with the same superscript letters mean that there is no significant difference at *P* < 0.05. LSD: least significant difference; NS: nonsignificant.

**Table 5:** Effect of treating diabetic rats with methanolic extracts of *N. sativa* and propolis for 4 weeks on water consumption.

Water consumed (mL/day)	Statistics	G1 N. control	G2 P. control	G3 *N. sativa *extract	G4 Propolis extract
1st week	Mean ± SE	33.33 ± 1.05^b^	42.50 ± 1.11^a^	36.33 ± 0.88^b^	36.33 ± 0.88^b^
	LSD				
	0.05 = 3.257				
	*t*-test	—	−4.56^*∗∗∗*^	7.40^*∗∗∗*^	4.01^*∗∗*^

2nd week	Mean ± SE	35.33 ± 1.17^b^	42.50 ± 1.11^a^	34.83 ± 0.90^b^	37.16 ± 0.79^b^
	LSD				
	0.05 = 2.943				
	*t*-test	—	−4.73^*∗∗∗*^	5.54^*∗∗∗*^	4.54^*∗∗∗*^

3rd week	Mean ± SE	29.16 ± 1.53^b^	42.50 ± 1.11^a^	26.66 ± 1.66^b^	26.66 ± 1.05^b^
	LSD				
	0.05 = 3.725				
	*t*-test	—	−8.00^*∗∗∗*^	7.88^*∗∗∗*^	19.00^*∗∗∗*^

4th week	Mean ± SE	27.50 ± 1.11^a^	29.16 ± 1.53^a^	28.00 ± 1.00^a^	27.50 ± 1.11^a^
	LSD				
	0.05 = 4.453				
	*t*-test	—	−1.58^NS^	0.63^NS^	1.00^NS^

Data are represented as mean ± SE. *t*-test values: *∗∗*: significant at *P* < 0.01 and *∗∗∗*: significant at *P* < 0.001. ANOVA analysis: within each row, means with different superscript (a, b, c, or d) are significantly different at *P* < 0.05, whereas means with the same superscript letters mean that there is no significant difference at *P* < 0.05. LSD: least significant difference; NS: nonsignificant.

**Table 6:** Effect of treating diabetic rats with methanolic extracts of *N. sativa* and propolis for 4 weeks on body weight gain (BWG) and food efficiency ratio (FER).

Biological evaluation	Statistics	G1 N. control	G2 P. control	G3 *N. sativa *extract	G4 Propolis extract
BWG (g/day)	Mean ± SE	0.494 ± 0.040^a^	0.466 ± 0.024^a^	0.316 ± 0.044^a^	0.444 ± 0.126^a^
	LSD	—	0.57^NS^	3.08^*∗∗*^	0.17^NS^
	0.05 = 0.191				
	*t*-test				

BWG (g/4 week)	Mean ± SE	14.833 ± 1.222^a^	14.000 ± 0.730^a^	9.500 ± 1.335^a^	13.333 ± 3.783^a^
	LSD				
	0.05 = 5.735				
	*t*-test	—	0.57^NS^	3.09^*∗∗*^	0.17^NS^

BWG%	Mean ± SE	8.305 ± 0.685^a^	7.059 ± 0.436^ab^	5.012 ± 0.889^b^	6.818 ± 1.988^a^
	LSD				
	0.05 = 2.178				
	*t*-test	—	1.52^NS^	2.15^*∗∗*^	0.12^NS^

FER (g/day)	Mean ± SE	0.030 ± 0.002^a^	0.029 ± 0.001^a^	0.020 ± 0.002^a^	0.028 ± 0.007^a^
	LSD				
	0.05 = 0.011				
	*t*-test	—	0.32^NS^	3.09^*∗∗*^	0.12^NS^

FER%	Mean ± SE	3.013 ± 0.248^a^	2.894 ± 0.151^a^	1.985 ± 0.279^a^	2.793 ± 0.792^a^
	LSD				
	0.05 = 1.199				
	*t*-test	—	0.40^NS^	2.99^*∗∗*^	0.12^NS^

Data are represented as mean ± SE. *t*-test values: *∗∗*: significant at *P* < 0.01. ANOVA analysis: within each row, means with different superscript (a, b, c, or d) are significantly different at *P* < 0.05, whereas means with the same superscript letters mean that there is no significant difference at *P* < 0.05. LSD: least significant difference; NS: nonsignificant.

## Abbreviations

AGEs: Advanced glycation end products
BUN: Blood urea nitrogen
BWG: Body weight gain
BWG%: Body weight gain percentage
CAT: Catalase
CML: N*ε*-(Carboxymethyl) lysine
Cr: Creatinine
CVD: Cardiovascular disease
FBG: Fasting blood glucose
FER: Food efficiency ratio
FER%: Food efficiency ratio percentage
G1: The first negative control group receiving a single tail vein injection of 0.1 mol/L citrate buffer
G2: The second positive control diabetic group intravenously injected with freshly prepared streptozotocin (60 mg/kg body weight) in a 0.1 mol/L citrate buffer (pH 4.5)
G3: The third group, diabetic rats as in G2 treated with 20% w/w* Nigella sativa* methanolic extract for 4 weeks
G4: The fourth group, diabetic rats as in G2 treated with 20% w/w propolis methanolic extract for 4 weeks
GST: Glutathione-S-transferase
IDDM: Insulin-dependent diabetes mellitus
Igs: Immunoglobulins
IL-6: Interleukin-6
MDA: Malondialdehyde
*N. sativa*: * Nigella sativa*
ROS: Reactive oxygen species
SOD: Superoxide dismutase
STZ: Streptozotocin.
